# Distinct roles of presynaptic dopamine receptors in the differential modulation of the intrinsic synapses of medium-spiny neurons in the nucleus accumbens

**DOI:** 10.1186/1471-2202-8-8

**Published:** 2007-01-19

**Authors:** Takeo Mizuno, Claudia Schmauss, Stephen Rayport

**Affiliations:** 1 Department of Psychiatry, Columbia University, 1051 Riverside Drive, Unit 62, New York, NY 10032, USA; 2 Department of Neuroscience, New York State Psychiatric Institute, 1051 Riverside Drive, Unit 62, New York, NY 10032, USA

## Abstract

**Background:**

In both schizophrenia and addiction, pathological changes in dopamine release appear to induce alterations in the circuitry of the nucleus accumbens that affect coordinated thought and motivation. Dopamine acts principally on medium-spiny GABA neurons, which comprise 95% of accumbens neurons and give rise to the majority of inhibitory synapses in the nucleus. To examine dopamine action at single medium-spiny neuron synapses, we imaged Ca^2+ ^levels in their presynaptic varicosities in the acute brain slice using two-photon microscopy.

**Results:**

Presynaptic Ca^2+ ^rises were differentially modulated by dopamine. The D1/D5 selective agonist SKF81297 was exclusively facilitatory. The D2/D3 selective agonist quinpirole was predominantly inhibitory, but in some instances it was facilitatory. Studies using D2 and D3 receptor knockout mice revealed that quinpirole inhibition was either D2 or D3 receptor-mediated, while facilitation was mainly D3 receptor-mediated. Subsets of varicosities responded to both D1 and D2 agonists, showing that there was significant co-expression of these receptor families in single medium-spiny neurons. Neighboring presynaptic varicosities showed strikingly heterogeneous responses to DA agonists, suggesting that DA receptors may be differentially trafficked to individual varicosities on the same medium-spiny neuron axon.

**Conclusion:**

Dopamine receptors are present on the presynaptic varicosities of medium-spiny neurons, where they potently control GABAergic synaptic transmission. While there is significant coexpression of D1 and D2 family dopamine receptors in individual neurons, at the subcellular level, these receptors appear to be heterogeneously distributed, potentially explaining the considerable controversy regarding dopamine action in the striatum, and in particular the degree of dopamine receptor segregation on these neurons. Assuming that post-receptor signaling is restricted to the microdomains of medium-spiny neuron varicosities, the heterogeneous distribution of dopamine receptors on individual varicosities is likely to encode patterns in striatal information processing.

## Background

Both schizophrenia and addiction are thought to involve aberrant information processing in the nucleus accumbens (nAcc), which makes up the ventral part of the striatal complex. In these disorders, changes in dopamine (DA) release apparently induce alterations in the intrinsic circuitry, affecting coordinated thought and motivation [1-3]. The cellular substrate for DA action in the striatal complex is remarkably homogeneous. Ninety-five percent of nAcc are medium-spiny GABAergic neurons (MSNs). MSNs receive extensive excitatory input from cortex, amygdala, hippocampus and thalamus, feed-forward GABAergic inhibition from a small population of fast-spiking interneurons, and excitation from large aspiny cholinergic neurons [4,5]. MSNs give rise to a profusion of local axon collaterals that ramify within their dendritic fields, as well as to all the efferent projections from the nucleus.

In somatic recordings from striatal MSNs, whose properties are nearly indistinguishable from those of nAcc MSNs, fast-spiking interneurons appear to be responsible for the majority of inhibitory input because they impinge on more proximal dendrites [5-7], while MSNs impinge on more distal dendrites and therefore appear deceptively weak [8-10]. Recent estimates have suggested that about two thirds of the GABAergic inputs to a given MSN are intrinsic connections from other MSNs [9,11]. Given both their number and probable strength, the intrinsic synapses of MSNs must have an important role in striatal information processing [9,10,12,13].

Considerable interest has focused on the targets of DA action in the striatal complex [14]. MSN-to-MSN synaptic connections show potent DA modulation. Guzman *et al. *[11] found that the intrinsic synapses were facilitated by D1 and inhibited by D2 agonists. However, Nicola *et al. *[15] and Taverna *et al. *[16] found the opposite for D1 agonists. Such contradictory results might reflect differences in the compliments of DA receptors on different MSN axons. In fact, the hypothesis that DA receptors might be differentially trafficked in the axon of MSNs was raised by Surmeier *et al. *[17] some time ago. However, all studies to date have been done with postsynaptic recording techniques that do not resolve single synaptic inputs. Thus, heterogeneity in the distribution of DA receptors has yet to be addressed directly.

To approach DA modulation at the level of individual synapses requires a way to activate and monitor the synapses of a single neuron. This becomes possible by using patch pipets both to fill single MSNs with a Ca^2+ ^indicator dye and then to stimulate them. Using this approach in combination with two-photon excitation laser scanning microscopy (2PLSM), which provides the requisite spatiotemporal resolution, we were able to monitor Ca^2+ ^transients at individual presynaptic varicosities as a proxy for synaptic action. We then used DA receptor-selective agonists and DA receptor knockout (KO) mice to elucidate the modulatory actions of DA. Our results support the hypothesis that MSNs differentially traffic DA receptors to presynaptic sites.

## Results

### Medium-spiny neuron intrinsic synapses function in the slice

Parasagittal slices were made through the striatal complex and whole cell recordings obtained from MSNs in the ventral part of the complex, or nucleus accumbens (nAcc). We isolated inhibitory connections using the glutamate antagonists CNQX (10 μM) and APV (50 μM). Overall 1,012 cells were recorded; of these, 253 cells had coplanar proximal axons with single or multiple hot spots of activity-dependent Ca^2+ ^responses. Of these, 38 cells remained healthy, with reproducible Ca^2+ ^responses such that the control and post-drug responses varied by less than 5% over the course of the experiment, making possible the acquisition of a full data set.

In preliminary experiments, we tested the synaptic function of MSN-to-MSN intrinsic synapses (see additional file 1). Since MSNs make all the projections from the striatal complex to the ventral pallidum (VP), a population of MSNs can be activated selectively by antidromic field stimulation in the VP [11,18]. This produced a complex IPSC, after a fixed delay of about 20 msec. Antidromic action potentials were occasionally evoked in the recorded cell (10% of cells), suggesting that some projection axons remained intact in our slice configuration. The delayed IPSC component was completely blocked by the GABA_A _antagonist bicuculline, and largely recovered, indicating that the intrinsic synapses of MSNs do indeed function, and inhibit neighboring MSNs.

### Two-photon imaging of axonal varicosities in the slice

**Figure 1:**
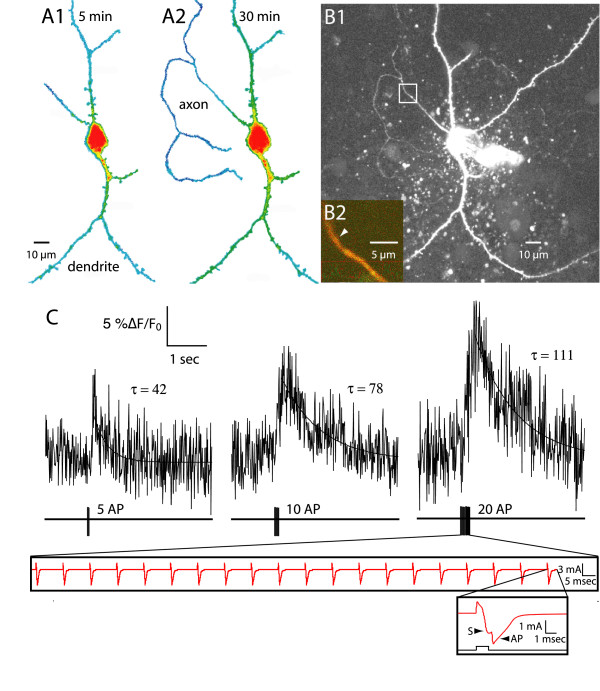
**Delayed dye diffusion identifies MSN axons**. A. A single MSN was loaded with OG1 and Alexa594 through the patch pipette and imaged by 2PLSM at 5 and 30 min after entering whole cell mode. Fluorescence intensity is shown in pseudocolor (ImageJ). Projection images (A1) were created from a z series of confocal Alexa594 images. At 5 min, the dendrites and proximal axon were filled. At 30 min (A2), dendritic labeling showed no significant change, while the axon was now clearly labeled. Dendritic processes were studded with spines, in contrast to the single axonal process, which was free of spines. B. The recorded MSN is shown in a projection image (B1) made from the full confocal z series of Alexa594 images (50 sections at 1 μm intervals). The area outlined in panel B1 (white rectangle) is shown enlarged in the inset (B2). This stretch of axon contained one hot spot, indicated by the arrowhead (B2). C. Presynaptic Ca^2+ ^responses were evoked by trains of 5, 10 or 20 AP's. The raw Ca^2+ ^responses showed the typical waveform of AP-induced Ca^2+ ^responses, with a fast rising and slow decay phase. The decay followed a single exponential curve; the decay time constant τ (in msec) is indicated on each panel. Raw traces of evoked AP's are shown in the bottom panel (red trace). Note that the 20 depolarizing pulses evoked 20 AP's without failures. The last AP is shown magnified in the inset (right bottom), with the depolarizing pulse below; the end of the stimulus artifact (S) is seen as an inflection preceding the unclamped axon spike (AP).

We patched MSNs with pipettes containing the Ca^2+ ^sensitive green fluorescent dye OG1 and the Ca^2+ ^insensitive red fluorescent dye Alexa594. MSN dendrites were easily recognized by virtue of their multiple spines, in contrast to the single non-spine bearing axon. Proximal dendrites and spines filled within 5 min of achieving whole cell mode, while the axon required at least 30 min to fill (Figure 1A), presumably because of its narrower bore. The single axonal process arose from the cell body and gave rise to several local axonal branches. One axonal branch extended beyond the confines of the dendritic tree to give rise to the efferent projection. In horizontal slices (n > 100), we never imaged axonal Ca^2+ ^rises, while in parasagittal slices we were successful in about 25% of experiments. Apparently, local MSN axons were more likely to be intact if the projection axon extended some distance without being cut. With the favorable orientation of the recorded cell in the slice, up to five varicose expansions or varicosities, could be imaged simultaneously in the same focal plane. We focused on proximal varicosities located within the second order branches, where dye concentrations stabilized within 30 min and provided reproducible signals.

In 3D reconstructions of recorded cells, the mean distance from the cell body to the recorded varicosities was 63 μm, with a range of 34–118 μm. Hot spots where Ca^2+ ^levels transiently increased with stimulation were distributed at irregular intervals along the axons and coregistered with varicose expansions – presumably synaptic varicosities – identified in the Alexa 594 images. These expansions had the typical *en passant *appearance of presynaptic varicosities on unmyelinated axons [19,20].

The major challenge in this study was recording Ca^2+ ^signals from the small synaptic varicosities of MSNs. Generally, axonal signals are several-fold dimmer than signals in small dendritic branches [20]. While Koester and Sakmann [21] were able to record single AP-induced Ca^2+ ^responses in the terminals of neocortical pyramidal cells, single AP-induced Ca^2+ ^responses were undetectable in MSN varicosities, presumably because they were embedded in the noise.

To detect Ca^2+ ^responses, we adjusted the Ca^2+^:Mg^2+ ^ratio to 3:0.5, 2:1 or 2:0 (in mM). Increasing the Ca^2+ ^concentration (ratio 3:0.5) yielded responses that became less reliable over time, presumably due to cytotoxicity. While normal Ca^2+ ^with reduced Mg^2+ ^(2:1) both enhanced the amplitude of the Ca^2+ ^responses and maintained reliability, the zero Mg^2+ ^ratio (2:0) further enhanced responses, without apparent detrimental effects, and was therefore used for subsequent experiments. However, adjusting the Ca^2+^:Mg^2+ ^ratio still not provide reliable single-AP evoked Ca^2+ ^responses, so we then moved to trains of depolarizing pulses. In a MSN with a single proximal axonal varicosity (Figure 1B), we found that AP-induced Ca^2+ ^responses (Figure 1C) increased with AP number. The Ca^2+ ^response showed the characteristic fast rise and slow decay kinetics [viz. [21,22]]. The decay followed a single exponential. In three MSNs, each with one varicosity, all three varicosities showed a maximal (plateau) response with 20 pulses. Trains of 10 pulses evoked sufficiently robust Ca^2+ ^responses that reached about 50% of the maximal level, and therefore provided the necessary dynamic range to measure both inhibitory and facilitatory effects. To minimize the contribution of the noise in the Ca^2+ ^measurements, we integrated the Ca^2+ ^responses (measuring the area under the curve, a.u.c.). Compared to measuring peak amplitudes, the integrated Ca^2+ ^response showed significantly less variability.

**Figure 2:**
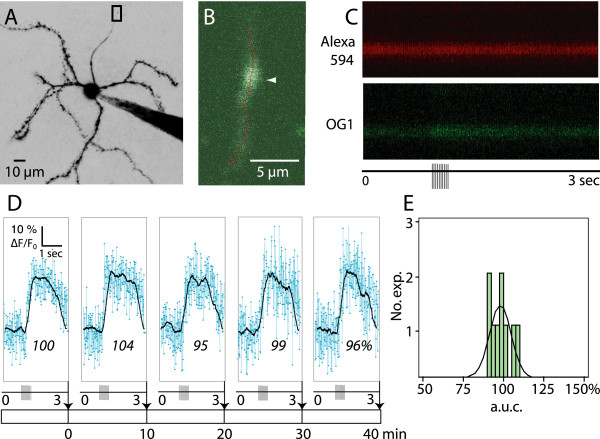
**Reproducible Ca^2+ ^responses at individual presynaptic varicosities evoked by brief tetanic stimulation**. A. The recorded MSN is shown as a projection image created from a confocal z series of Alexa594 images. The area outlined (black rectangle) corresponds to the area imaged in panel B. B. OG1 fluorescence image showing the stretch of axon contained one hot spot (arrowhead). The spline used is shown in red, superimposed on the OG1 image. C. Spline scan images. Top image shows Alexa594, and bottom OG1. The timing of repetitive depolarizing pulses to evoke 10 action potentials is indicated on the time line (bottom). As compared to the Alexa594 trace, the OG1 trace shows a stimulation-dependent increase in fluorescence intensity, which is shown in the line graphs in the next panel. D. Ca^2+ ^imaging was repeated under control conditions (without drug application) at 10 min intervals, as diagramed on the time line. Raw traces are shown in blue, and smoothed traces (moving average of ten, see Methods) are superimposed in black. The total duration of the experiment was 40 min. The magnitude of the subsequent Ca^2+ ^responses (measured as area under the curve, a.u.c.) expressed as a percent of the initial response is indicated below each trace. The Ca^2+ ^responses varied less than 5%. E. To test reproducibility, Ca^2+ ^responses were evoked twice under control conditions (no drugs) and the a.u.c. of the second response was expressed as the percent of the first response (n = 9). The second response did not differ significantly from the first (see Results).

To demonstrate reproducibility of Ca^2+ ^responses measured in this way, we did repeated stimulation under control conditions (without DA agonists), as shown in Figure 2. The recorded MSN is shown in a projection image with the stretch of the axonal process with a single varicosity outlined (Figure 2A). The corresponding OG1 image (Figure 2B) shows that the Ca^2+ ^response was spatially restricted to a short stretch that corresponded to the varicosity. A spline was superimposed (red line) on the OG1 image. The laser was repeatedly directed along the spline (spline scan) to produce a temporal display of the OG1 signal in response to a train of APs (Figure 2C). There was no change in the Ca^2+^-insensitive Alexa594 fluorescence.

In one experiment, we measured Ca^2+ ^responses with repeated stimulation at 10 min intervals, which showed the relative stability of responses under optimal conditions (Figure 2D). To test this further, we evoked a second, control Ca^2+ ^response in several cells. The second response was 98.0 ± 2.2% (mean ± s.e.m.) of the first, control response (Figure 2E). The distribution of second Ca^2+ ^responses did not differ significantly from the normal distribution (Shapiro-Wilk W test gave a W value of 0.93, with p = 0.47, which was not significant). Nor was it skewed (skewness was 0.44, which was less than the criterion of one standard error of 2.2). Based on this analysis, changes of 5% or greater were deemed significant. Pharmacological data were then classified as showing inhibition (response < 95% of control), no change (95–105%), or facilitation (>105%). Data were analyzed for modulatory actions of single agonists in experiments where Ca^2+ ^responses were stable for at least 20 min, and for multiple agonists in cells where Ca^2+ ^responses were stable for 40 min. Invariably somatic action potentials triggered Ca^2+ ^responses in all imaged varicosities, consistent with prior observations [20,21,23].

### DA modulation of individual MSN varicosities in wild type mice

**Figure 3:**
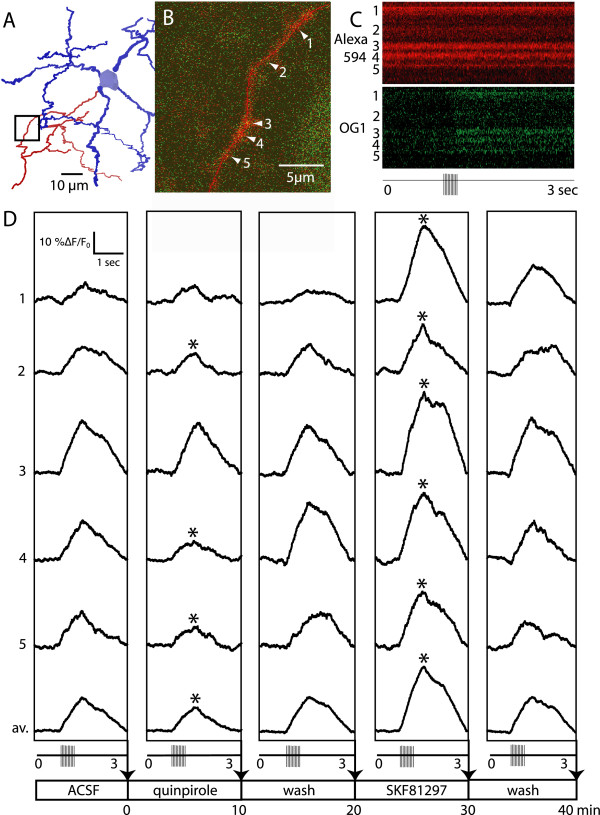
**D1- and D2 modulation of individual varicosities on the same axonal segment**. A. The MSN imaged is shown in a 3D reconstruction of a confocal z series of Alexa594 images. The cell body and multiple spiny dendrites are shown in blue and the single non-spine bearing axon is shown in red. The area imaged is outlined (black rectangle) and shown in panel B. B. In the OG1 fluorescence image, the stretch of axon that contained five hot spots (numbered). The spline used for the time scan is shown as a red line superimposed on the axonal segment. C. The spline scan images show no change in the Alexa594 fluorescence and a stimulus-dependent increase in OG1 fluorescence. D. Ca^2+ ^imaging was carried out at 10 min intervals. The total duration of the experiment was 40 min. Asterisks identify Ca^2+ ^responses that were significantly modulated by DA (> 5% of control). Note that the majority of varicosities showed modulation with both D1 and D2 agonists. The relative magnitude of modulation was strikingly heterogeneous.

We used the D1/D5 selective agonist SKF81297 (1 μM) and the D2/D3 selective agonist quinpirole (20 μM) to examine DA modulation of single MSN synapses. In Figure 3, the recorded MSN was rendered into a 3Dimage, with the axon shown in red, the dendrites in blue and the scanned area outlined (Figure 3A). The corresponding OG1 image (Figure 3B) showed that the stretch of axon contained five presynaptic varicosities. The spline used for scanning is shown superimposed on the axon. The spline scan images showed that the Ca^2+ ^responses or hot spots (Figure 3C, OG1 traces) corresponded to varicosities (Figure 3C, Alexa594 traces). Hot spots #2, 4 and 5 showed the representative DA response (Figure 3D), namely inhibition with quinpirole (40–77% of control), and facilitation with SKF81297 (116–309%). Hot spots #1 and 3 did not respond to quinpirole, but showed clear facilitation with SKF81297.

**Figure 4:**
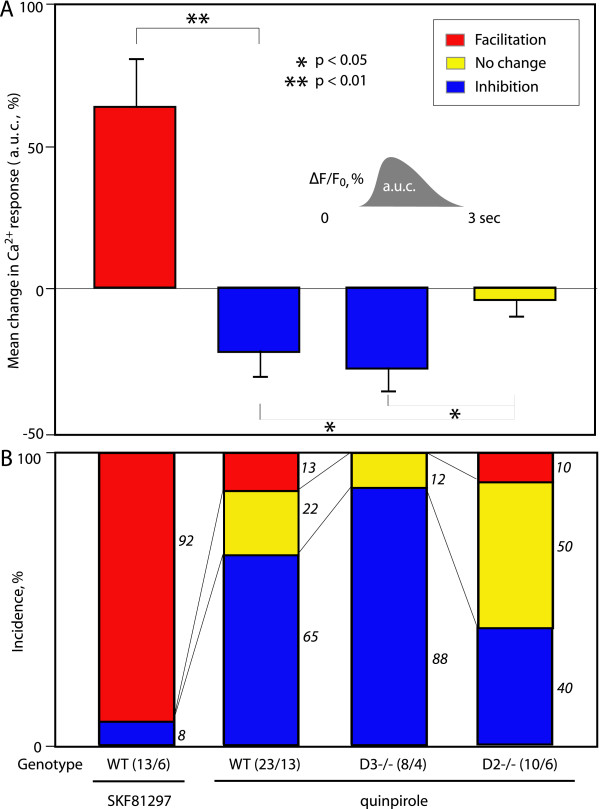
**Summary of DA receptor agonist modulation in wild type and DA receptor KO mice**. A. The mean change in Ca^2+ ^responses is shown for the full data set (bars are colored to indicate main effect). Asterisks mark significant differences. The quinpirole inhibition seen in WT and D3 KO mice was completely abolished in D2 KO mice (p < 0.05). B. The incidence of DA modulation was assessed using the 5% criterion (see text). Numbers in parentheses indicate the total number of varicosities and cells recorded, respectively. While D1 stimulation with SKF81297 produced facilitation in almost all instances, D2 stimulation with quinpirole produced both inhibition and facilitation. Quinpirole facilitation seen in WT and D2 KO mice was completely abolished in D3 KO mice.

As summarized in Figure 4, we recorded 13 varicosities from 6 cells with SKF81297 and 23 varicosities from 13 cells with quinpirole in wild type mice (the greater variability in the quinpirole results required the additional experiments). Overall, SKF81297 produced facilitation (166 ± 17% of control), while quinpirole produced inhibition (77 ± 9%) (Figure 4A). As shown in the incidence data (Figure 4B), SKF81297 nearly always produced facilitation, while quinpirole produced predominantly inhibition, but in some instances facilitation, and in others it had no effect.

### Studies in DA receptor knockout mice

**Figure 5:**
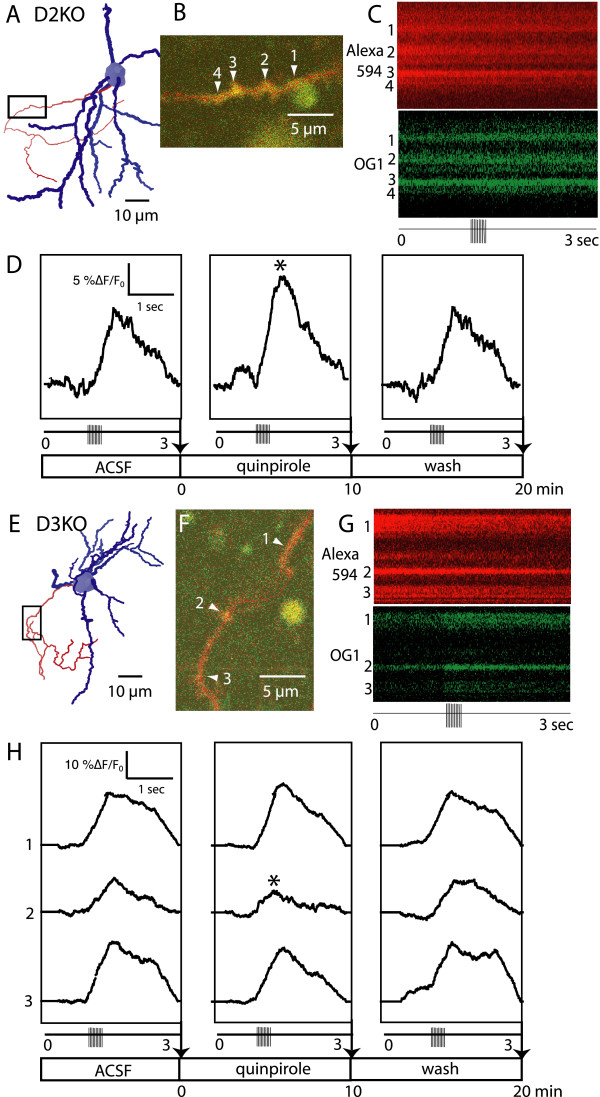
**Dissection of quinpirole effects using D2 and D3 KO mice**. A. A 3D reconstruction of a MSN from a D2 KO mouse is shown with the cell body and dendrites in blue and the axon in red. The area outlined (black rectangle) corresponds to the area imaged in panel B. B. The OG1 image contained four hot spots (numbered). C. Spline scan image. Top panel shows Alexa594, and bottom panel shows OG1. In the OG1 image, only varicosity #4 showed a Ca^2+ ^response. D. In this D2 KO cell, quinpirole facilitated the Ca^2+ ^response. E. 3D reconstruction of a MSN from a D3 KO mouse. The area outlined (black rectangle) corresponds to the area imaged in panel F. F. The area imaged contained three hot spots (numbered). G. Spline scan image. Top panel shows Alexa594, and bottom panel shows OG1. H. In this D3 KO cell, quinpirole inhibited varicosity #2 (*), but had no effect on varicosities #1 and 3.

Because quinpirole activates both D2 and D3 receptors [24], the heterogeneity in quinpirole modulation might reflect differential effects at D2 and D3 receptors. To address this, we made slices from D2 single, D3 single and D2/D3 double knockout (KO) mice [25]. In a D2 KO mouse (Figure 5A–D), the area imaged contained a single hot spot that showed robust quinpirole facilitation (164% of control; Figure 5D), presumably D3 receptor-mediated. In a D3 KO mouse (Figure 5E–H), the area imaged contained 3 hot spots. Hot spot #2 showed significant inhibition (53% of control), presumably D2 receptor-mediated, while #1 and 3 showed no effect (Figure 5H).

As also summarized in Figure 4, quinpirole inhibition was apparently normal in D3 KO mice, while it was largely abolished in D2 KO mice (Figure 4A), suggesting that the predominant inhibitory effect was D2 receptor-mediated. In D2 KO mice, the incidence of quinpirole inhibition was diminished (Figure 4B), while the no effect incidence increased. However, quinpirole-mediated inhibition remained in D2 KO mice, suggesting that D3 receptors must also mediate inhibition. While the incidence of quinpirole-induced facilitation remained almost the same in D2 KO mice, it was undetectable in D3 KO mice. Thus, D2 receptors appeared to mediate inhibition, while D3 receptors appeared to mediate either inhibition or facilitation. The incidence of quinpirole-mediated inhibition was slightly increased in D3 KO mice, which may reflect a compensatory increase in D2 receptor expression due to the lack of D3 receptors, as D2 and D3 receptors may exert feedback control on their expression [25].

### Heterogeneity in DA modulation

**Figure 6:**
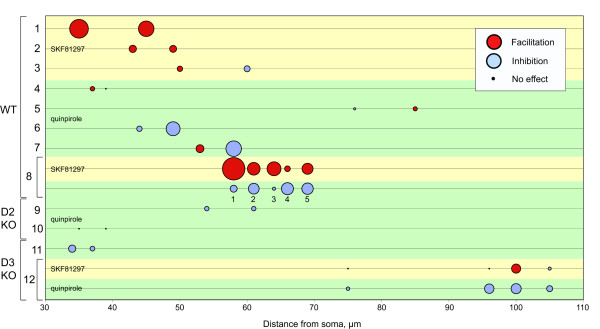
**DA receptor-mediated modulation is heterogeneous**. Bubble plot of spatial distribution of varicosities and magnitude of DA receptor-mediated modulation in wild type and D2 and D3 KO mice. The distance of imaged varicosities from the soma is indicated along the x-axis. Bubble area is proportional to the magnitude of modulation. Facilitation is shown in red and inhibition in purple. In experiments two experiments, both SKF81297 and quinpirole were tested sequentially in the same cell (experiments 8 and 12). In experiment 8 (data shown in Figure 3), the majority of varicosities showed both D1 receptor-mediated facilitation and D2 receptor-mediated inhibition, but the magnitude of modulation varied strikingly among individual varicosities. In experiment 12, in a D3 KO mouse, the third varicosity showed both SKF81297-mediated facilitation and quinpirole-mediated inhibition, indicating colocalization of D1 and D2 receptors.

The correlation between the magnitude of SKF81297 or quinpirole-mediated modulation and the distance from soma to the recorded varicosities was subjected to regression analysis (see additional file 2). For both SKF81297 and quinpirole application, the data were randomly distributed, showing no relationship to distance from the soma. In the experiments where we were able to image more than one varicosity on the same axon (n = 12), heterogeneity in DA modulation was readily apparent. To look for a spatial pattern more closely, we graphed DA modulatory effects at individual varicosities as a function of their distance from the cell body in a bubble plot (Figure 6). In experiment 8, where we tested both SKF81297 and quinpirole in wild type mice, hot spots #1 and #3 showed robust SKF81297-mediated facilitation but weak quinpirole-mediated inhibition, while #4 showed robust quinpirole-mediated inhibition and weaker SKF81297-mediated facilitation. Varicosity #2 and the most distal varicosity showed equally robust SKF81297-mediated facilitation and quinpirole-mediated inhibition. In experiment 12, where we tested both SKF81297 and quinpirole in a D3KO mouse, the most proximal hot spot was unaffected, while the second showed only quinpirole-mediated inhibition, and the third showed both SKF81297-mediated facilitation and quinpirole-mediated inhibition. Thus, the relative responsiveness to D1 and D2 receptor activation varied considerably from varicosity to varicosity along the axon, with no proximal-distal pattern.

## Discussion

We examined DA modulation of medium-spiny neuron synapses by visualizing activity-dependent Ca^2+ ^responses at multiple presynaptic varicosities on proximal axons of individual MSNs. Ca^2+ ^responses showed potent DA modulation, with subsets of varicosities showing both D1 and D2 pharmacology. D1 receptors were almost exclusively facilitatory, while D2 receptors were inhibitory, and D3 receptors either inhibitory or facilitatory. Along single axonal processes DA modulation varied strikingly from varicosity to varicosity.

### Modulatory role of presynaptic DA receptors

The D1 agonist SKF81297 produced reliable facilitation of presynaptic Ca^2+ ^responses, while the D2/D3 agonist quinpirole produced either facilitation or inhibition. This was consistent with our previous cell culture observations [26], as well as recent observations in isolated striatal synaptosomes [27]. Due to the lack of D3 receptors in the dorsal striatum [28], the inhibition of synaptic currents with a D2/D3 agonist observed by Guzman *et al. *[11] was presumably mediated by D2 receptors. In the nAcc, where D3 receptors are robustly expressed, dissecting D2 actions becomes more complicated.

Given the limited selectivity of available D2 agonists, one approach might be to use DA in combination with more selective than DA antagonists [29]. However, there are potential confounds in such an approach, including instability of DA, differential accessibility of DA and DA antagonists to receptors in the slice and limited reversibility of antagonists due to their lipophilicity. [30-33]. We therefore opted to use D2 and D3 KO mice. In D2 KO mice, the inhibitory effect of quinpirole was diminished, indicating that D2 receptors mediate inhibition. The facilitatory effect of quinpirole was eliminated in D3 KO mice, while it remained in D2 KO mice, indicating that D3 receptors mediate facilitation. However, some inhibition remained in D2 KO mice, indicating that D3 receptors may mediate either facilitation or inhibition.

We can discount a role for D4 receptors in the modulation of nAcc MSN synapses. Results from *in situ hybridization *[28,34] and immunohistochemistry [35,36], which reveal D3 receptor expression in about 20% of MSNs, show negligible D4 receptor expression. No D4 receptor expression was seen in the striatum or nAcc of *Drd4*-EGFP transgenic mice, in which enhanced green fluorescent protein (EGFP) was introduced using bacterial artificial chromosome (BAC) technology and expressed under the control of genomic fragments flanking the D4 receptor gene locus [37]. Electron microscopic immunohistochemistry revealed some D4 receptor expression in dendritic spines of a minority of MSNs [38], consistent with physiological evidence that D4 actions are postsynaptic [29]. In two preliminary experiments in D2/3 double KO mice (not shown), quinpirole had no effect, confirming that effects of quinpirole are mediated by D2 or D3 receptors, but not D4 receptors. Thus, D4 receptors are unlikely to have a role in the presynaptic modulation of MSNs.

D3 receptors may mediate more heterogeneous actions as they are not robustly coupled to G_i _in many mammalian expression systems [39]. Furthermore, the third intracellular loop, which is important for the G protein coupling, is highly divergent in D3 receptors with only a ~25% homology in amino acid sequence to the D2 sequence, suggesting a divergence in coupling. For instance, phosphorylation of G protein coupled receptors can switch them from G_s _to G_i_-coupled in an activity-dependent fashion [40]. It is thus possible that the coupling of the D3 receptor is not only more heterogeneous, but probably plastic.

### Co-localization of D1 and D2 receptors with distinct functions on intrinsic synapses

Although the dominant view of DA receptor expression in striatal neurons asserts strict cellular segregation [41,42], several studies [26,43-51] have suggested that D1- and D2-like receptors are co-expressed in single MSNs. Here, we have shown that subsets of presynaptic varicosities D1 pharmacology, others D2, and many both D1 and D2 pharmacology. This suggests a resolution to these apparently contradictory findings – DA receptors may be differentially trafficked to different presynaptic varicosities in MSN axons [17,26]. As D1 receptors mediate facilitatory effects while D2-like receptors mediate predominantly inhibitory effects, DA could be either facilitatory or inhibitory – with varying potency – depending on the relative preponderance of functional D1 and D2-like receptors on individual varicosities.

### DA differentially modulates intrinsic synapses

The DA agonists triggered strikingly divergent effects at different varicosities, including adjacent varicosities on the same axon, presumably reflecting heterogeneity in the relative abundance of functional DA receptors on individual varicosities. In light of accumulating evidence that the distribution of many receptors is dynamically regulated by recruitment to postsynaptic sites, internalization and differential recycling [52], it seems plausible that the distribution of DA receptors on individual presynaptic varicosities might also vary considerably. In fact, this appears to be the case for MSN projection axons. Anatomical tracing studies have confirmed that all MSNs project to the pallidum, with about one third terminating there, and the remaining two thirds extending to the entopeduncular nucleus and the ventral midbrain [53,54]. Physiological studies have shown that these projections are differentially modulated by DA; projections to pallidal neurons are inhibited by D2 receptor activation [55], while projections to the ventral midbrain are facilitated by D1 receptor activation [56,57]. These observations can be reconciled if D1 and D2 receptors are coexpressed in MSN cell bodies but differentially trafficked in the axons of a given MSN [17].

Several lines of evidence support the idea that DA receptors undergo differential trafficking. Alternatively spliced D2 receptor isoforms (D2S and D2L) are differentially targeted to different presynaptic terminals, where they mediate distinct functions [58]. Consistent with this, we found in D3KO mice that the effect of quinpirole was heterogeneous, indicating that D2 receptors are heterogeneously distributed. Alternatively, DA receptors might be uniformly trafficked to all presynaptic terminals, but differentially distributed between surface (functional) and intracellular (nonfunctional) compartments. For example, D2 receptors are selectively sorted to degradation pathways, while D1 receptors are preferentially recycled back to the surface [59]. Consequently, synapses expressing DA receptors may have an altered expression profile depending on prior DA exposure. This suggests that the differential distribution of DA receptors observed at a given moment might reflect past patterns of DA release.

### Microdomains in DA receptor-mediated signaling

How could the observed DA receptor-mediated modulation be so spatially restricted? Voltage-dependent Na^+ ^or K^+ ^channels are modulated directly by DA [43,60,61], which should control presynaptic Ca^2+ ^influx. But this is unlikely to account for the micro-heterogeneity we observed, since the varicosities recorded were often quite close together and were therefore probably isopotential. In contrast, cAMP which was in earlier studies thought to rapidly diffuse through the cytosol [62,63], has been found in more recent studies, using sensitive biosensors, to be restricted in its diffusion to microdomains. [64-66]. Molecules diffuse more slowly in the cytosol due to the molecular crowding effect [67], which would be slowed further by the confinement effect of the narrow bore of axon. Restricted signaling might be further ensured by the recruitment of active enzymes into close proximity of Ca^2+ ^channels through the interaction with scaffold proteins. [68-70]. For instance, in dendritic spines, Ca^2+ ^rises are modulated by G protein-coupled receptors through the activation of protein kinase A in a spatially restricted manner [71]. Thus, the assembly of a multimeric signaling complex together with restricted diffusion of second messengers should enable local DA modulation of nearby Ca^2+ ^channels.

Synaptic vesicle release is triggered by clustered presynaptic Ca^2+ ^channels that produce discrete domains of elevated intracellular Ca^2+^. Among the members of the Cav2 calcium channel family that mediate the presynaptic Ca^2+ ^rise, N-type and P/Q-type channels are found on synapses interconnecting MSNs [72], and are subject to DA modulation [73]. Due to cooperativity amongst clustered Ca^2+ ^channels, the channels may function as a single Ca^2+ ^entry site [74]. But because of the non-linear relationship between Ca^2+ ^influx and vesicle release, modest modulatory effects on individual Ca^2+ ^channels should translate into more dramatic changes in transmitter release, while at the same time restricting modulatory effects to subcellular microdomains [75]. Thus, the DA modulation of Ca^2+ ^responses we observed could control GABA release at individual MSN varicosities and therefore play a role in encoding striatal information processing.

## Conclusion

The present results raise the intriguing hypothesis that the differential distribution of DA receptors reflects past patterns of DA synaptic activity, and may thus be a novel site for enduring synaptic plasticity. Intrinsic connections may be viewed as the hidden layer in a Hopfield network [76] – so their modulation would be expected to shape information flow through the area and confer greater computational capacity for motor and reward-related learning. The multiplicity of DA receptor actions in combination with their differential distribution could produce large variations in DA responsiveness at individual synapses. Conceivably, aberrant patterns of DA receptor activation could induce pathologic changes in the distribution of DA receptors, giving rise to disorganized information flow, and ultimately to aberrant motor or appetitive behaviors.

## Methods

### Slice preparation

All animal procedures were reviewed and approved by the Institutional Animal Care and Use Committees of Columbia University and the New York State Psychiatric Institute and were in compliance with the USPHS *Guide for the Care and Use of Laboratory Animals*. Slices were prepared from postnatal day 11–13 congenic C57BL/6 and 129 wild type or congenic C57BL/6 D2, D3 or D2/D3 receptor knockout (KO) mice [25] following standard procedures. Animals were deeply anesthetized with ketamine/xylazine, and their brains rapidly removed into ice-cold artificial cerebrospinal fluid (ACSF Recipe1, in mM: 125 NaCl, 2.5 KCl, 25 NaHCO_3_, 1.25 NaH_2_PO_4_, 2 CaCl_2_, 1 MgCl_2_, 25 glucose; pH 7.4) saturated with 95% O_2_-5% CO_2_. Using a vibratome, 300 μm thick parasagittal slices were cut encompassing the projection from the nucleus accumbens (nAcc) to the adjacent ventral pallidum (VP). After 1 hour recovery, slices were placed in the recording chamber (about 1 ml capacity) on the stage of an upright fluorescence microscope (LSM510, Carl Zeiss MicroImaging, Thornwood, NY) and held down with a custom-made harp made from platinum wire. During recording and imaging, the recording chamber was continuously perfused with ACSF (Recipe2: 125 NaCl, 2.5 KCl, 25 NaHCO_3_, 1.25 NaH_2_PO_4_, 3 CaCl_2_, 0 MgCl_2_, 25 glucose; pH7.4) saturated with 95% O_2_-5% CO_2 _at about 1 ml/min. Recordings were done at ~20°C, to optimize data acquisition; working at room temperature increased stability in both the recording and imaging, and improved the temporal resolution by slowing the response kinetics.

### Whole cell patch clamp recording

Using IR-DIC microscopy, visually-guided whole cell recordings were obtained with patch pipettes filled with an intracellular solution (130 KCl, 4.6 MgCl_2_, 10 HEPES, 4 ATP·Na_2_, 0.4 GTP·Na, pH 7.25) containing 100 μM Oregon Green 488 BAPTA-1 (OG1) and 10 μM Alexa 594 (Molecular Probes/Invitrogen, Carlsbad, CA). Patch pipettes were fabricated from standard wall borosilicate glass capillary tubes with filament (Harvard Apparatus, Holliston, MA) and had access resistances of 4–8 MΩ. During dye loading (30 min), access resistance typically increased to about 13–15 MΩ, probably due to partial resealing, and then remained constant for the rest of the experiment. Cells were voltage clamped at -70 mV, without series resistance compensation, using an Axopatch 200 amplifier (Axon Instruments/Molecular Devices, Sunnyvale, CA) coupled to an ITC-18 A/D interface (InstruTech, Port Washington, NY). Generation of pulse patterns and monitoring of whole cell currents were done with PulseControl XOPs [Richard J. Bookman, University of Miami School of Medicine, Department of Medical Network Services, Miami, FL, [77]] running under IgorPro (WaveMetrics, Lake Oswego, OR). Stimulus parameters were adjusted so that depolarizing steps (50 mV, 1 msec) reliably evoked action potentials without failure.

### Two-photon calcium imaging

Once in whole-cell mode, the fluorescent dyes were allowed to diffuse into cells and their processes for a minimum of 30 min or until Alexa594 fluorescence reached a plateau in proximal varicosities. Dye diffusion was followed in sequential images taken at ~10 min intervals. The recorded cell was imaged to confirm the identification of the cell as a MSN according to morphological criteria, specifically cell body diameter of ~10 μm (medium-sized) and the presence of multiple spiny dendrites. A single non-spine-bearing axonal process was readily discerned, after the longer time necessary for diffusion into the axon due to its narrower bore (see Figure 1A).

2PLSM Ca^2+ ^imaging procedures followed established methodology [20,78,79]. We used a Zeiss LSM510 equipped with a direct-coupled Ti:sapphire laser (Mira Model 900F, Coherent, Santa Clara, CA) tuned to 850 nm (excitation wavelength). Presynaptic Ca^2+ ^transients evoked by repetitive action potentials propagation were recorded by monitoring the fluorescence intensity of OG1. Cells were stimulated with a brief high-frequency train (ten 1 msec depolarizing pulses at 50 Hz, for 200 msec) to evoke ten action potentials without failure. This produced a constant, submaximal (unsaturated) Ca^2+ ^response. Changes in the Ca^2+ ^signal, %ΔF/F_o _= %(F-F_o_)/F_o _(where F_o _= average of OG1 fluorescence intensity before delivery of depolarization pulses), were measured at 10 msec intervals for 3 sec in line-scan mode. TTL trigger pulses were sent from Igor Pro to synchronize the acquisition of membrane current traces, line-scan images and the train stimulation. Ca^2+ ^rises were typically restricted to short brighter stretches or swellings of axons identified in the Alexa 594 images, which were well suited for line scanning [19,21].

To define a free shape curve (spline) as the scan line, a mouse-drawn line was superimposed on well-focused stretches of axon containing multiple varicosities, and saved and reused for subsequent imaging. However, if the contour of the defined spline was too tortuous, the scanner path was artifactually displaced with respect to the spline. To compensate for this, the X, Y offset was adjusted to bring the scanner line back into register with the spline. If this was not sufficient, then the spline was simplified so that the scanner followed the spline exactly. The LSM software generated a profile of fluorescence intensity along the spline on which hot spots (points with higher fluorescence intensity) were readily identified. Their locations along the axon were measured in the 3D reconstruction generated from z-series of Alexa 594 images using Neurolucida and Neurolucida Explorer (MicroBrightfield, Williston, VT).

### Drug application

The selective D1/D5 agonist SKF81297 (1 μM) or the D2/D3 agonist quinpirole (20 μM) were applied via bath perfusion. Drug effects took about 5 min to reach a plateau (or wane), so we measured Ca^2+ ^responses at 10 min intervals. To avoid residual drug effects, we recorded from only one cell in each slice.

### Data analysis

Acquired data were analyzed with LSM Examiner software (Carl Zeiss MicroImaging) and ImageJ [Wayne S. Rasband, National Institutes of Health, Bethesda, MD, [80]]. Changes in the Ca^2+ ^signal were calculated as %ΔF/F_o_, which normalizes for slow shifts in baseline fluorescence [19,79]. The area under the curve (a.u.c., integration) of unmodified raw traces was calculated using Igor Pro. Representative Ca^2+ ^responses were smoothed by a moving average method using Igor Pro, in which each data point was replaced by the average of the 10 preceding and 10 following points. Statistical comparisons were made using JMP (SAS Institute, Cary, NC). To detect the difference between two independent comparison groups, an appropriate *t *test was chosen based on the result of the Ftest. When the result of the Ftest indicated that the variances of the comparison groups were not significantly different (p > 0.05)), the two-tail student *t *test was used. When the result of the Ftest indicated that the variances of the comparison groups are significantly different (p < 0.05), Welch's *t *test was used. Graphs were created using Igor Pro and JMP.

## Authors' contributions

TM designed and conducted the experiments. CS suggested the use of D2/D3 KO mice to dissect the D2 pharmacology, provided D2, D3 single and D2/D3 double KO mice, and critical insights throughout. SR conceived and directed the project. TM and SR wrote the manuscript. All three authors read and approved the final manuscript.

## Supplementary Material

Additional File 1 **Functional intrinsic synapses**. Electrophysiological demonstration of functional intrinsic synapses formed by MSNs in the parasagittal brain slices. We found that making parasagittal slices, in which the projection axons of MSNs extending from nAcc to ventral pallidum (VP) were most likely to be intact, was crucial for recording Ca^2+ ^responses from the intrinsic synapses. Subsets of projection axons were stimulated with a bipolar electrode placed in the VP (schematized in left panel; arrow in right panel indicated the stimulus artifact), according to the previously established protocol [11,18]. Spikes in MSN axons conducted antidromically to the soma in the nAcc and then orthodromically into their local axons to activate their intrinsic synapses. This produced a complex IPSC (right panel, top trace) that was completely blocked by the GABA_A _antagonist bicuculline (middle trace). Following wash, the drug effect partially reversed (bottom trace). Thus, MSN intrinsic synapses are functional in our slice preparation. File is in pdf format. Click here for file

Additional File 2 **Spatial correlation of dopamine modulation**. Scatter diagram of spatial correlation between the magnitude of D1 (SKF81297) or D2/D3 (Quinpirole) modulation (expressed as the percent of the preceding control response) and the distance from soma to the recorded varicosities (μm). The sample correlation coefficient (Pearson Correlation Coefficient) was denoted as r. For both SKF81297 (left) and quinpirole (right), data were randomly distributed with weak linearity. File is in pdf format. Click here for file
