# Supplementary figures and images for: Distinct roles of presynaptic dopamine receptors in the differential modulation of the intrinsic synapses of medium-spiny neurons in the nucleus accumbens
Source: BMC Neurosci. 2007 Jan 19;8:8. doi: 10.1186/1471-2202-8-8 (PMC1783657; doi:10.1186/1471-2202-8-8)

A1

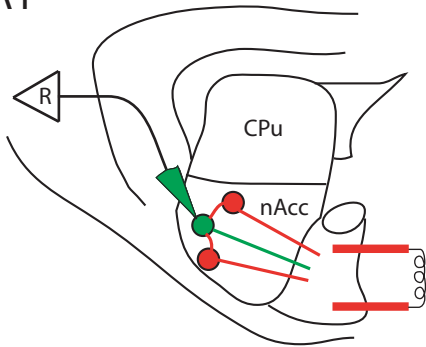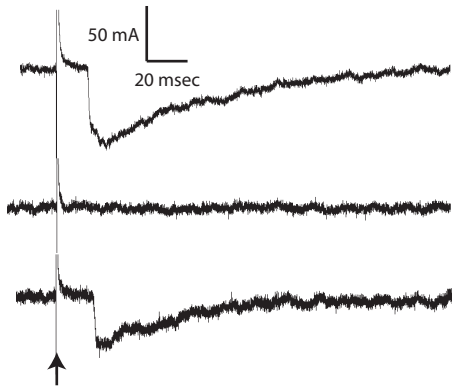

Supplement: Additional File 1 — Functional intrinsic synapses. Electrophysiological demonstration of functional intrinsic synapses formed by MSNs in the parasagittal brain slices. We found that making parasagittal slices, in which the projection axons of MSNs extending from nAcc to ventral pallidum (VP) were most likely to be intact, was crucial for recording Ca2+ responses from the intrinsic synapses. Subsets of projection axons were stimulated with a bipolar electrode placed in the VP (schematized in left panel; arrow in right panel indicated the stimulus artifact), according to the previously established protocol [11,18]. Spikes in MSN axons conducted antidromically to the soma in the nAcc and then orthodromically into their local axons to activate their intrinsic synapses. This produced a complex IPSC (right panel, top trace) that was completely blocked by the GABAA antagonist bicuculline (middle trace). Following wash, the drug effect partially reversed (bottom trace). Thus, MSN intrinsic synapses are functional in our slice preparation. File is in pdf format. [file 1471-2202-8-8-S1.pdf]

# A2

## SKF81297

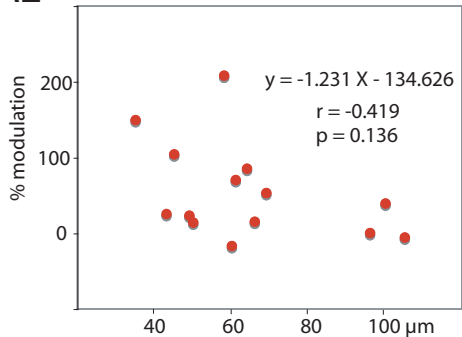

## quinpirole

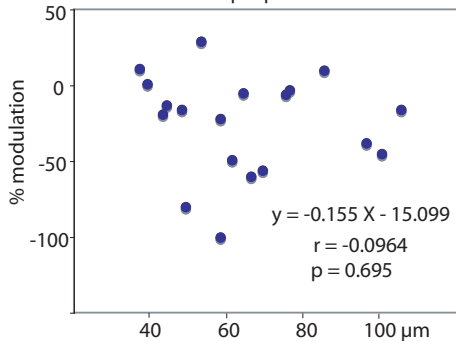

Supplement: Additional File 2 — Spatial correlation of dopamine modulation. Scatter diagram of spatial correlation between the magnitude of D1 (SKF81297) or D2/D3 (Quinpirole) modulation (expressed as the percent of the preceding control response) and the distance from soma to the recorded varicosities (μm). The sample correlation coefficient (Pearson Correlation Coefficient) was denoted as r. For both SKF81297 (left) and quinpirole (right), data were randomly distributed with weak linearity. File is in pdf format. [file 1471-2202-8-8-S2.pdf]
